# Research gaps identified during systematic reviews of clinical trials: glass-ionomer cements

**DOI:** 10.1186/1472-6831-12-18

**Published:** 2012-06-29

**Authors:** Steffen Mickenautsch

**Affiliations:** 1 SYSTEM Initiative, Department of Community Dentistry, Faculty of Health Sciences, University of the Witwatersrand, 7 York Rd, Parktown, Johannesburg, 2193, South Africa

## Abstract

**Background:**

To report the results of an audit concerning research gaps in clinical trials that were accepted for appraisal in authored and published systematic reviews regarding the application of glass-ionomer cements (GIC) in dental practice

**Methods:**

Information concerning research gaps in trial precision was extracted, following a framework that included classification of the research gap reasons: ‘imprecision of information (results)’, ‘biased information’, ‘inconsistency or unknown consistency’ and ‘not the right information’, as well as research gap characterization using PICOS elements: population (*P*), intervention (*I*), comparison (*C*), outcomes (*O*) and setting (*S*). Internal trial validity assessment was based on the understanding that successful control for systematic error cannot be assured on the basis of inclusion of adequate methods alone, but also requires empirical evidence about whether such attempt was successful.

**Results:**

A comprehensive and interconnected coverage of GIC-related clinical topics was established. The most common reasons found for gaps in trial precision were lack of sufficient trials and lack of sufficient large sample size. Only a few research gaps were ascribed to ‘Lack of information’ caused by focus on mainly surrogate trial outcomes. According to the chosen assessment criteria, a lack of adequate randomisation, allocation concealment and blinding/masking in trials covering all reviewed GIC topics was noted (selection- and detection/performance bias risk). Trial results appear to be less affected by loss-to-follow-up (attrition bias risk).

**Conclusion:**

This audit represents an adjunct of the systematic review articles it has covered. Its results do not change the systematic review’s conclusions but highlight existing research gaps concerning the precision and internal validity of reviewed trials in detail. These gaps should be addressed in future GIC-related clinical research.

## Introduction

Systematic reviews are defined, according to the Cochrane collaboration, as scientific literature reviews aimed at answering clearly formulated questions through use of systematic and explicit methods for identifying, selecting and critically appraising relevant research, and for collecting and analysing data from the literature included in the review [1]. Through this process, systematic reviews provide a unique opportunity for also identifying gaps in the currently available research on a particular topic and providing recommendations for addressing these. In order for such objective to become part and parcel of the regular systematic review process, it has been suggested that the identification of research gaps should follow a systematic approach that is based on widely accepted elements and provides transparent and reproducible results [2].

Robinson et al. (2011) identified the lack of a systematic process for the development of ‘future research’ sections in systematic reviews and developed a framework for determining research gaps [2]. The framework included a classification of research gap reasons and research gap characterization, using the PICOS elements: population (*P*), intervention (*I*), comparison (*C*), outcomes (*O*) and setting (*S*). Reasons for research gaps are classified as ‘imprecision of information (results)’, ‘biased information’, ‘inconsistency or unknown consistency’ and ‘not the right information’. In addition, a worksheet with instructions for facilitating the use of such framework during or after systematic reviews was developed [2].

In order to systematically identify potential research gaps in clinical trials on the topic of glass-ionomer cements (GIC) in dentistry, an audit of all authored and previously published GIC-related systematic reviews [3-10] was conducted on the basis of recommendations by Robinson et al. (2011) [2]. All systematic reviews were conducted and published between March 2010 and March 2011 on clinical topics covering the application of conventional (C-GIC) and resin-modified GIC (RM-GIC). Although each contained recommendations for future research in their discussion section, these were not derived on the basis of a systematic framework.

Therefore, the purpose of this article is to provide an addition to authored and previously published GIC-related systematic reviews by reporting on the results of an audit concerning identified research gaps in clinical trials covering the application of glass-ionomer cements in dental practice.

## Materials and method

All authored systematic reviews published on the topic of glass-ionomer cement (GIC) [3-10] were selected and reviewed in relation to aspects of the precision and internal validity of the accepted clinical trials.

Information concerning trial precision was extracted, following recommendations by Robinson et al. regarding a framework for identifying research gaps during systematic reviews [2]. In the context of this audit, the following classification was adopted for investigating research gaps in the precision of clinical trials:

(i) Imprecision of results: e.g. confidence intervals (95% CI) wide enough to suggest both superiority and inferiority at the same time; no, or too few trials found; too small sample size of clinical trials accepted in systematic reviews [2,11,12].

(ii) Inconsistency/unknown consistency of results: e.g. conflicting directions of effect sizes; magnitude of differences in effect sizes and significance of such differences; non-overlapping confidence intervals (95% CI); unexplained clinical and/or statistical heterogeneity; lack of sufficient trials (at least two trials are needed in order to assess consistency) [2,11].

(iii)  Lack of right information: e.g. only surrogate outcomes investigated; follow-up period too short [2].

PICOS characteristics of research gaps included information (if available) on (*P*): age, gender, ethnicity and clinical patient characteristics; (*I*) and (*C*): specific name(s) of treatment(s); (*O*): the measured/indented clinical outcomes (*S*): type of clinical setting. All extracted information was entered into a modified abstraction worksheet. Modifications of the worksheet included:

(i) Exclusion of ‘Bias information’ (assessment for systematic error/bias risk was undertaken separately in more depth than per the original recommendations);

(ii) Inclusion of all gap reasons, instead of only the most important one (thus addressing one of the identified weaknesses of the framework) [2];

(iii)  Sub-grouping of clinical topics per systematic review (where appropriate).

The internal validity of clinical trials was assessed through three of the systematic reviews [3,4,7], following recommendations by Berger [13]. Such assessment of internal trial validity is based on the understanding that successful control for systematic error cannot be assured on the basis of inclusion of adequate methods alone; i.e. adequate generation of random sequence, allocation concealment, blinding/masking, as part of the trial methodology. Such inclusion, and even the adequate reporting of such inclusion by following the CONSORT statement [14], merely indicates an attempt to control adequately against systematic error. In addition, empirical evidence is required as part of the trial report, from which it may be ascertained that such an attempt was indeed successful. Without such evidence, internal validity of clinical trials cannot be assured [13].

This form of assessment was not yet adopted in five systematic reviews [5,6,8-10]. Thus all accepted clinical trials of these were re-assessed, using the more recent criteria (Table 1). All results of the internal validity assessment were collated with each clinical topic per systematic review.

**Table 1:** Quality assessment criteria of trials

**Selection bias**		
**Score**	**Criteria**	**Impact on bias risk**
**Randomisation and concealment**		
A	Randomisation: Details of any adequate type of allocation method that generates random sequences, with the patient as unit of randomization, are reported.^1^	Doubts may still exist about whether the trial results are influenced by selection bias but no indication can be found in the trial report to support such doubt.
	Concealment: Trial provides evidence^2^ that concealment was indeed effective and that the random sequence could not have been observed or predicted throughout the duration of the trial.	
B	Randomisation: Details of any adequate type of allocation method that generates random sequences, with the patient as unit of randomisation,are reported.^1^	Despite the implementation of a method considered able to prevent unmasking of the concealed allocation sequence through direct observation and prediction, there are reasons to expect that the concealed allocation sequence may have been unmasked during the course of the trial.
	Concealment: Trial reports on any adequate method for preventing direct observation^3^ and prediction^4^ of the allocation sequence and sequence generation rules.	
C	Randomisation: Details of any adequate type of allocation method that generates random sequences, with the patient as unit of randomization, are reported.^1^	Despite the implementation of a method considered able to prevent unmasking of the concealed allocation sequence through direct observation, there are reasons for expecting that operators could have predicted the concealed allocation sequence.
	Concealment: Trial reports on any adequate method to prevent direct operator observation of allocation sequence and sequence generation rules^3^. However, the allocation sequence and sequence generation may have been sufficiently predicted.	
D	Randomisation: Details of any adequate type of allocation method that generates random sequences, with the patient as unit of randomization, are reported.^1^	Despite the theoretical chance for each patient to be allocated to either treatment group, operator knowledge of the allocation sequence may have led to patient allocation that favoured the outcome of one type of treatment above the other.
	Concealment: The trial report does not include information on how the allocation of random sequence was concealed. The allocation could have been directly observed and/or predicted.	
0	Trial does not comply with criteria A – D.	No guarantee of equal chance for patients to be allocated to either treatment group. Thus allocation may have favoured the outcome of one type of treatment above the other.
Baseline data for randomised trials		
A	Baseline data collected before randomisation and reported for both treatment groups. Data shows no significant differences between both groups.	Evidence is given that randomisation has led to equal groups, suggesting little risk of selection bias.
B	Baseline data collected before randomisation and reported for both treatment groups. Data shows significant differences between both groups but has been appropriately statistically adjusted.	Differences have been adjusted. Thus the influence of possible selection bias appears to be reduced.
C	Baseline data collected before randomisation and reported for both treatment groups. Data shows significant differences between both groups without being statistically adjusted.	Reported differences may be due to ineffective randomisation, thus indicating risk of selection bias.
0	Trial does not comply with criteria A – C.	No evidence is given as to whether randomisation has indeed led to equal groups with differences beyond chance. Thus differences may exist, indicating selection bias.
**Detection/Performance bias**		
**Blinding/Masking**		
**Score**	**Criteria**	**Impact on bias risk**
A	Trial reports on any type of method that is known to prevent patient AND operator AND evaluator from discerning whether patients are allocated to the test- or the control group (Blinding/Masking).	Evidence is given that the trial results may not have been influenced by detection/performance bias which may have favored the outcome of one type of treatment above the other.
	Trial reports a process by which the effect of Blinding/Masking was evaluated, as well as the results of such evaluation.	
B	Trial reports on any type of method that is known to prevent patient AND operator AND evaluator from discerning whether patients are allocated to the test- or the control group (Blinding/Masking).	Doubts may still exist about whether the trial results are influenced by detection/performance bias but no indication can be found from the trial report to support such doubt. However, no evaluation of the blinding/masking effect has been included in the trial. Thus no evidence for lack of bias is given.
	Trial report does not give reason for doubt that the patient allocation to either the test- or the control group has been unmasked throughout the duration of the trial.	
C	Trial reports on any type of method that is known to prevent patient AND operator AND evaluator from discerning whether patients are allocated to the test- or the control group (Blinding/Masking).	Despite the implementation of a method considered able to prevent unmasking, there are reasons to expect that operators/patients could have discovered the allocation.
	Trial report gives reason for doubt that the patient allocation to either the test- or the control group has been unmasked throughout the duration of the trial.	
0	No process able to blind/mask patients AND operators as to whether patients were allocated to either the test- or the control group reported or implemented (It is insufficient to report that blinding/masking was done without reporting the details of the process).	Knowledge about the patient allocation may have caused patients/operator to act in a way that may have favoured the outcome of one type of treatment above the other,
**Attrition bias**		
**Loss – to follow up**		
**Score**	**Criteria**	**Impact on bias risk**
A	Available case analysis, loss-to-follow-up reported per treatment group. Subsequent sensitivity analysis does not indicate a possible risk of bias.	The trial allows extraction of evidence that attrition may not have favoured the outcome of one type of treatment above the other.
B	Available case analysis, loss-to-follow-up reported per treatment group. Subsequent sensitivity analysis indicates a possible risk of bias.	The trial allows assessment of the risk that attrition may have favoured the outcome of one type of treatment above the other.
0	Trial does not report number of included participants per treatment group at baseline or gives any indication that would allow ascertaining of the loss-to-follow up rate per treatment group.	The trial carries an unknown risk that attrition may have favoured the outcome of one type of treatment above the other.

^1^ Excluded are types of allocation methods that are considered as inadequate: cluster randomisation, fixed block randomisation with block size 2, minimisation, alternation, randomisation of teeth, use of date of birth or patient record number, “quasi”-randomisation, split-mouth.

^2^ E.g. by reporting results of the Berger-Exner Test or any other statistical tests that show that covariates of compared groups were similar at baseline.

^3^ E.g. by opening of opaque envelope, obtaining allocation from tables, computer generated or from other sources.

^4^ E.g. central randomisation, sequence allocation by other than operator; excluding varied block randomisation.

## Results

### Research gaps in the precision of clinical trial results

Extracted information indicative of existing research gaps concerning the precision of clinical trial results related to GIC are shown in Table 2. The most common reasons for gaps are found in the ‘Imprecision of results – A’, followed by ‘Inconsistency of results – B’. These are due to a general lack of clinical trials; insufficient sample size in available trials and wide 95% confidence intervals, as well as to large differences in effect sizes, unexplained existing he-terogeneity of data and the difficulty in judging consistency from single trials, respectively (Table 3). Only few research gaps have been ascribed to ‘Lack of information – C’; i.e. assessment of mainly surrogate, instead of clinical out-comes.

**Table 2:** Research gaps abstraction worksheet related to trial precision

**SR**	**T**	**Reason for Gap**			**Population [P]**	**Intervention [I]**	**Comparison [C]**	**Outcome [O]**	**Setting [S]**
		**A**	**B**	**C**					
Mickenautsch and Yengopal, 2011 [3]	[a]		x		Children, with permanent dentition Age 6–18 years, boys and girls, from various countries in Asia, Europe, South America	C-GIC based	Resin-based fissure sealant	Caries on pits and fissures	Standard dental clinical settings (Improvised dental setting at school premises in one trial)
						fissure sealant			
Mickenautsch and Yengopal, 2011 [4]	[a]	x			Children, with permanent dentition Age 11–16 years, boys and girls, from Tanzania, Sweden, Syria	C-GIC tooth	Amalgam tooth restorations	Caries on restoration margins	Standard dental clinical settings (Improvised dental setting with portable equipment at a shaded open-air area in one trial)
						restorations			
	[b]	x			Children, with primary dentition Age 5–13 years, boys and girls, from various countries in Asia and Europe	C-GIC tooth	Amalgam tooth restorations	Caries on restoration margins	
						restorations			
Mickenautsch et al., 2010 [5]	[a]	x			Patients with Class V restorations in permanent teeth	ART restorations	Amalgam tooth restorations	Restoration success	Standard dental clinical settings
	[b]				Patients with Class I restorations in permanent teeth	ART restorations	Amalgam tooth restorations	Restoration success	
	[c]	x	x		Patients with Class II restorations in permanent teeth	ART restorations	Amalgam tooth restorations	Restoration success	
	[d]				Patients with Class I restorations in primary teeth	ART restorations	Amalgam tooth restorations	Restoration success	
	[e]	x			Patients with Class II restorations in primary teeth	ART restorations	Amalgam tooth restorations	Restoration success	
Mickenautsch and Yengopal, 2010 [6]	[a]	x	x	x	Children, with permanent dentition Age 12–17 years, boys and girls,	RM-GIC restorations	Composite restoration (F)	Caries lesions on adjacent teeth	Standard dental clinical settings
	[b]	x	x	x		RM-GIC restorations	Composite restoration (NF)	Caries lesions on adjacent teeth	
Yengopal and Mickenautsch, 2011 [7]	[a]	x	x		Orthodontic patients with fixed appliances on permanent teeth	RM-GIC bonding	Composite bonding (NF)	Caries lesions around orthodontic brackets	Standard dental clinical settings
	[b]	x	x		Patients with restorations in primary teeth	RM-GIC tooth	Composite restoration (F)	Caries on restoration margins	
						restorations			
	[c]	x	x		Patients with restorations in primary teeth	RM-GIC tooth	Composite restoration (NF)	Caries on restoration margins	
						restorations			
	[d]	x	x		Patients with restorations in permanent teeth	RM-GIC tooth	Composite restoration (F)	Caries on restoration margins	
						restorations			
	[e]	x	x		Patients with restorations in permanent teeth	RM-GIC tooth	Composite restoration (NF)	Caries on restoration margins	
						restorations			
Yengopal and Mickenautsch, 2011 [8]	[a]				Patients with permanent teeth	RM-GIC based fissure sealant	Resin-based fissure sealant	Caries free pits and fissures	Standard dental clinical settings (Improvised dental setting with portable equipment in one trial)
Mickenautsch et al., 2010 [9]	[a]				Patients with restorations in primary teeth	C-GIC tooth restorations	RM-GIC tooth restorations	Caries free restoration margins	Standard dental clinical settings
	[b]	x			Patients with restorations in permanent teeth	C-GIC tooth restorations	RM-GIC tooth restorations	Caries free restoration margins	
Mickenautsch et al., 2010 [10]	[a]	x		x	Patients with deep cavities in permanent teeth	RM-GIC tooth restorations	CaOH tooth restorations	Pulp response	Standard dental clinical settings
						in deep cavities	in deep cavities		

*SR* = Systematic review, *A* = Imprecision of results; *B* = Inconsistency of results; *C* = Lack of right information; *GIC* = Glass-ionomer cement; *RM-GIC* = Resin-modified GIC; *C-GIC* = Conventional GIC; *ART* = Atraumatic restorative treatment (C-GIC based); *(F)* = Containing fluoride; *(NF)* = Not containing fluoride; *T* = Topic; *CaOH* = Calcium hydroxide cement.

**Table 3:** Details of identified research gaps in clinical topics related to trial precision

**Research gap**	**Details**
Imprecision of results [A]	Too few clinical trials
	Caries on restoration margins on permanent teeth: C-GIC tooth restorations vs. Amalgam tooth restorations [4]
	Caries on restoration margins on primary teeth: C-GIC tooth restorations vs. Amalgam tooth restorations [4]
	Success of restorations in permanent teeth: ART vs. Amalgam tooth restorations (Class II), (Class V) [5]
	Success of Class II restorations in primary teeth: ART vs. Amalgam tooth restorations [5]
	Caries lesions on adjacent permanent teeth: RM-GIC vs. Composite restorations (F), (NF) [6]
	Carious lesions around orthodontic brackets: RM-GIC vs. Composite bonding (NF) [7]
	Caries on restoration margins on primary teeth: RM-GIC vs. Composite restorations (F), (NF) [7]
	Caries on restoration margins on permanent teeth: RM-GIC vs. Composite restorations (F), (NF) [7]
	Caries-free restoration margins on permanent teeth: C-GIC vs. RM-GIC restorations [9]
	Pulp response on teeth with deep caries: RM-GIC vs. CaOH cement [10]
	Sample size too small
	Caries on restoration margins on primary teeth: C-GIC tooth restorations vs. Amalgam tooth restorations [4]
	Success of restorations in permanent teeth: ART vs. Amalgam tooth restorations (Class II), (Class V) [5]
	Carious lesions around orthodontic brackets: RM-GIC vs. Composite bonding (NF) [7]
	Caries on restoration margins on permanent teeth: RM-GIC vs. Composite restorations (NF) [7]
	Pulp response on teeth with deep caries: RM-GIC vs. CaOH cement [10]
	Confidence intervals (95% CI) wide enough to include superiority and inferiority
	Caries-free restoration margins on permanent teeth: C-GIC vs. RM-GIC restorations [9]
Inconsistency of results [B]	Contradicting directions of effect sizes/Large differences between effect sizes
	Caries on pits and fissures of sealed permanent teeth: C-GIC vs. resin-based sealants [3]
	Carious lesions around orthodontic brackets: RM-GIC vs. Composite bonding (NF) [7]
	Caries on restoration margins on permanent teeth: RM-GIC vs. Composite restorations (NF) [7]
	Unexplained heterogeneity
	Caries on pits and fissures of sealed permanent teeth: C-GIC vs. resin-based sealants [3]
	Only 1 trial found – no assessment of consistency possible
	Success of restorations in permanent teeth: ART vs. Amalgam tooth restorations (Class II) [5]
	Caries lesions on adjacent permanent teeth: RM-GIC vs. Composite restorations (F), (NF) [6]
	Caries on restoration margins on primary teeth: RM-GIC vs. Composite restorations (F), (NF) [7]
	Caries on restoration margins on permanent teeth: RM-GIC vs. Composite restorations (F) [7]
Lack of right information [C]	Only/mostly surrogate outcomes accessed
	Caries lesions on adjacent permanent teeth: RM-GIC vs. Composite restorations (F), (NF) [7]
	Pulp response on teeth with deep caries: RM-GIC vs. CaOH cement [10]

*GIC* = Glass-ionomer cement; *RM-GIC* = Resin-modified GIC; *C-GIC* = Conventional GIC; *ART* = Atraumatic restorative treatment (C-GIC based); *(F)* = Containing fluoride; *(NF)* = Not containing fluoride; *T* = Topic; *CaOH* = Calcium hydroxide cement.

The result of the PICOS assessment (Table 2) shows that in total 19 different clinical topics were appraised through the eight systematic reviews [3-10]. Of these, nine topics related to interventions (*I*) using RM-GIC and eight to C-GIC (of which five were related to ART implementation). Two topics comprised clinical comparisons (*I* and *C*) of both GIC classes with each other, concerning the permanent and primary dentition (*P*) with regard to caries in restoration margin (*O*). The clinical work of most reviewed trials was performed in standard dental clinical settings *(S).*

The comparison (*C*) for seven of the eight C-GIC topics included amalgam restorations and one resin-based pit and fissure sealant as controls. The subject population (*P*) of the C-GIC topics were patients with permanent (5 topics) and primary (3 topics) dentition. The outcomes (*O*) were related to caries on pits and fissures, caries on restoration margins and restoration success rates for one, two and five topics, respectively.

RM-GIC was compared (*C*) against composite resin (with and without fluoride), resin-based fissure sealant material and calcium hydroxide cement in seven, one and one topics, respectively. The outcomes (*O*) were related to caries on pits and fissures (1 topic), caries on restoration margins (4 topics), caries around orthodontic brackets (1 topic), caries on adjacent teeth (2 topics) and pulp response (1 topic). The studied population (*P*) comprised patients with permanent (6 topics) and primary (3 topics) dentition.

Table 3 provides further information as to the specific clinical topics affected by the identified research gaps. Gaps were identified in regard to clinical comparisons of C-GIC and RM-GIC versus amalgam, composite resin (with or without fluoride) and calcium hydroxide cement to clinical outcomes, related to caries on restoration margins as well pits and fissures, restoration success rates, orthodontic bracket bonding failures and pulp response in deep cavities.

### Research gaps in the internal validity of clinical trial results

Table 4 presents the results of the assessment of systematic error/bias risk affecting the internal validity of clinical trial results. This table shows the number of datasets judged according to the chosen assessment criteria (Table 1). Only criteria “A” indicate adequacy. According to the chosen assessment criteria, a complete lack of adequate randomisation, allocation concealment and blinding/masking in all the reviewed GIC topics can be noted. Trials related to the topics of GIC-based fissure sealants and GIC (including ART) comparison to amalgam restorations included limited evidence for equivocal baseline characteristics between intervention groups. Trial results for most GIC topics appear to be less affected by loss-to follow up (attrition bias).

**Table 4:** Assessment of systematic error/bias risk of clinical trials per topic

SR	T	DS	Selection bias risk									Detection/Performance				Attrition bias risk		
												Bias risk						
			Randomisation and					Baseline data comparison				Blinding/				Loss-to- follow -up		
			Allocation concealment					between groups				Masking						
			A	B	C	D	0	A	B	C	0	A	B	C	0	A	B	0
Mickenautsch and Yengopal, 2011 [3]	[a]	30					30	9			21				30	18	7	5
Mickenautsch and Yengopal, 2011 [4]	[a]	12					12	2			10				12	10	2	
	[b]	5					5	1			4				5	3	2	
Mickenautsch et al., 2010 [5]	[a]	1					1	1							1			1
	[b]	12					12	9			3				12	3		9
	[c]	6					6	6							6			6
	[d]	6				1	5	1			5				6			6
	[e]	2				1	1	1			1				2			2
Mickenautsch and Yengopal, 2010 [6]	[b]	12					12				12				12	12		
Yengopal and Mickenautsch, 2011 [7]	[a]	4					4				4				4	4		
	[b]	2					2				2				2	1	1	
	[c]	5					5				5				5	5		
	[d]	4					4				4				4	4		
	[e]	9					9				9				9	6	3	
Yengopal and Mickenautsch, 2010 [8]	[1]	19					19				19				19	19		
Mickenautsch et al., 2010 [9]	[a]	8					8				8				8	1	7	
	[b]	14					14				14				14	14		
Mickenautsch et al., 2010 [10]	[a]	18					18				18				18	18		

*SR* = Systematic review; *T* = Topic; DS = Total number of datasets per topic.

Adequate = Score A; Inadequate = Scores B, C, 0.

## Discussion

This is the first attempt to adopt a framework for determining research gaps in systematic reviews [2] on a specific subject in dentistry. This attempt is limited to authored systematic review articles concerning GIC only, and may thus not reflect the totality of potentially existing research gaps regarding GIC. However, one of the challenges reported by the team that developed the framework was that they were not involved with the conduct of the evidence review and the writing of its results [2]. In seeking to avoid any negative impact of such challenge, it was decided that only authored systematic reviews relevant to GIC should be selected and other systematic reviews based on a systematic literature search on the GIC topic would not be included. For that reason, the present audit should not be considered in a stand-alone capacity, but as an adjunct and partial update of the systematic review articles [3-10] it has covered. As the scope of this audit did not affect reported effect sizes and overall values of established internal validity, its results do not change the systematic reviews’ conclusions. Instead, the audit results give empirical support to the general recommendations, already made in the original systematic review reports [3-10], for further research needs in terms of precision and validity.

### Identified research gaps in the precision of clinical trial results

The PICOS framework in Table 2 shows a comprehensive and interconnected coverage of GIC-related clinical topics that were appraised through the eight systematic reviews [3-10]: Topics focused on the two relevant clinical classes of glass-ionomer cement (GIC): conventional GIC and resin-modified GIC (RM-GIC). Both material classes are related to each other but also differ in aspects of clinical indication and biocompatibility. Hence, systematic review evidence was appraised for both in terms of anticariogenic effects regarding preventive, i.e. pit and fissure protection [3,4] and restorative indications [4,6,7]. In addition, evidence for RM-GIC was appraised for its orthodontic indication [7] and claims of its potential pulp toxicity were reviewed [10]. Further evidence regarding comparison of the potential anticariogenic effects of GIC and RM-GIC [9] was assessed. With the development of the atraumatic restorative treatment approach (ART), on a C-GIC basis, current clinical evidence concerning the overall success rate of ART fillings in comparison to amalgam as prevailing gold standard was included [5].

The results presented in the modified abstraction worksheet suggest a general lack of trials on many GIC-related topics as the main reason for research gaps (Table 2). The same topics suffer from weak statistical power due to small sample sizes of available trials and related to wide confidence intervals that render their results inconclusive (Table 3).

A particular need for more trials of suitable sample size has been identified for clinical topics concerning results that were identified as statistically significant: the longevity (restoration success rate) of Class II and V ART restorations in permanent teeth, in comparison to that of amalgam fillings placed with the use of traditional high-speed drills. Systematic review results concerning Class II and V restorations identified a statistically significant improvement of longevity favouring ART by 28% over amalgam after 6.3 years, and by 61% after 2.3 years, respectively [5]. However, according to the findings in this audit (Table 2 and 3), the precision of these results are to be regarded as low and thus future studies are needed in order confirm or reject the initial results.

In a meta-analysis comparing C-GIC and amalgam restorations, amalgam restorations were found to have a 65% higher chance than C-GIC restorations of having caries on restoration margins after 6 years [4]. Although this difference in effect size was reported to be statistically significant (p = 0.001), its precision is limited, owing to the small number of trials (n = 2) included.

### Identified research gaps in the internal validity of clinical trial results

According to the chosen assessment criteria (Table 1), none of the clinical trials accepted for further appraisal in the eight systematic reviews [3-10] included adequate randomisation/allocation concealment or blinding/masking, thus suggesting a high risk of selection and detection/performance bias (Table 4). Some of the trials from three systematic reviews [3-5] reported on the lack of statistically significant differences between selected covariates of the test- and control groups at baseline. Such lack of significant difference means a lack of evidence that randomisation was ineffective. However, this may not provide enough evidence that randomisation was effective either, as other non-measured and/or unknown covariates may exist that differ significantly between groups as a result of potential lack of effective randomisation. Hence, reasons for doubt remain regarding this point and risk of selection bias may still be high.

Bias or systematic error constitutes any factor in the knowledge acquisition process that systematically diverts its outcomes away from true values [15]. Systematic error, therefore, limits the internal validity of acquired knowledge and thus renders all clinical results included in this audit - even those for which no research gaps in terms of precision were identified (Table 2 and 3) - in need for confirmation by higher quality future trials.

Meta-epidemiological studies have attempted to investigate and quantify the bias risk resulting from lack of adequate randomisation/allocation concealment or blinding/masking. The empirical evidence of these studies was appraised by a Cochrane systematic review in regard to randomisation and allocation concealment [16]. Its conclusions affirmed that the effect of not using randomisation could be as large as, or larger than, the expected effects of intervention, and therefore recommend the use of random allocation and adequate allocation concealment. However, the identified evidence for this conclusion was identified as weak [16]: larger effect estimates were found in non-randomised trials than in randomised studies of the same intervention and measured outcome. This is in agreement with the suggestion that effect-overestimation due to bias is likely to be most common [17]. However, the results originated from only one single meta-epidemiological study [18] and were based on analysis of vote counting, with no quantitative estimates of effect [16]. Other studies that investigated randomised versus non-randomised trials of different interventions and/or measured outcomes provided overall only inconsistent and conflicting evidence [16]. More studies that consider the influence of heterogeneity due to differences in clinical intervention and outcome measures are needed in order to provide better evidence concerning the impact of potential bias risk on clinical trial results.

In the absence of strong empirical evidence of bias, e.g. due to lack of adequate randomisation, arguments for correcting potential bias impact still rely largely on logical considerations. Berger [13] has added to the debate by presenting the logical consideration that successful control for bias cannot be assured on a basis of inclusion of adequate methods, (adequate generation of random sequence, allocation concealment, blinding/masking) alone. Such inclusion would only indicate an attempt to control bias adequately but additionally requires evidence, as part of the trial report, from which it may be ascertained that such an attempt was indeed successful. Without such evidence, so it is argued, high internal validity of clinical trials cannot be assured [13]. Even though the empirical basis for this argument remains weak, the initial evidence [16] appears to support its adoption into the methodology of randomised controlled trials (RCT). Such adoption would also have the benefit of generating a volume of specific trials that would be available for future comparison in meta-epidemiological studies: by comparing the outcome of RCTs, which have adopted the above considerations with those that have not. An empirical investigation still provides the best method for conclusively answering questions concerning adequate bias control. Against this background, the following recommendations may be made for future GIC-related RCTs:

(i) Inclusion into the trial report of a detailed description of the randomisation process, methods and restriction, as well as detailed description of the allocation concealment process;

(ii) Inclusion into the trial methodology of any possible form of quantitative test that may identify the presence of selection bias; e.g. test for association of the trial end point with the probability P{E} that a certain patient receives a certain treatment (test- or control intervention), as well as inclusion of such test results into the trial report;

(iii)  Inclusion into the trial report of a detailed description of any form of blinding/masking attempt;

(iv)  Inclusion into the trial methodology of any possible form of quantitative test that may identify the presence of detection/performance bias; e.g. appraisal of the opinions of patients and independent evaluators as to which type of treatment a patient may have received, followed by statistical comparison of such data with the actual allocated treatment sequence, as well as inclusion of the test results into the trial report.

## Conclusions

This audit identified research gaps in the precision and internal validity of clinical trials on topics regarding the application of glass-ionomer cements. Gaps concerning precision are mainly manifest in the insufficient trial number and insufficient sample size. Gaps concerning internal validity are related to uncertainties of potential bias risk due to inadequate randomisation, concealment of the random allocation, as well as blinding/masking. It is recommended that these research gaps may be addressed by focussing in future randomised controlled trials on the identified reasons.

## Competing interests

The author contributes to the conduct and publication of systematic reviews concerned with topics related to Minimum Intervention (MI) in dentistry.

## Author’s contribution

SM developed the concept and outline and wrote this paper. The author read and approved the final manuscript.

## Pre-publication history

The pre-publication history for this paper can be accessed here:

http://www.biomedcentral.com/1472-6831/12/18/prepub
